# Case Report: Repigmentation and complete hair regrowth in an 11-year-old preadolescent with alopecia totalis treated with a JAK inhibitor

**DOI:** 10.3389/fped.2026.1867141

**Published:** 2026-07-16

**Authors:** Yanhong Jia, Xue Wang, Yuanxiang Liu, Zigang Xu, Bin Zhang

**Affiliations:** Department of Dermatology, National Center for Children’s Health, Beijing Children’s Hospital, Capital Medical University, Beijing, China

**Keywords:** alopecia areata, baricitinib, preadolescent, repigmentation, ritlecitinib

## Abstract

Alopecia areata (AA) is an autoimmune disease characterized by non-scarring hair loss. Janus kinase (JAK) inhibitors have emerged as effective therapeutic options for severe pediatric AA, though repigmentation of white hair during treatment remains rarely reported. Herein, we report an unusual case of complete hair regeneration with simultaneous white-to-black hair conversion in a child with severe AA following JAK inhibitor therapy. An 11-year-old girl presented with alopecia totalis (AT) persisting for two years. She initially received baricitinib for seven months, achieving SALT 65% with predominantly white and gray hair regrowth. After switching to ritlecitinib 50 mg/day, black hair emerged at 6 weeks, with complete hair regrowth and repigmentation at 6 months (SALT 0). The patient has now completed 12 months of ritlecitinib therapy with sustained response and stable pigmentation. Family Dermatology Life Quality Index (FDLQI) decreased from 18 to 4. No significant adverse effects were observed. This case suggests that prolonged JAK inhibition may facilitate both hair regrowth and pigment restoration in pediatric severe AA.

## Introduction

Alopecia areata is an autoimmune disorder characterized by non-scarring hair loss (1).However, the treatment of severe alopecia areata, especially alopecia totalis and alopecia universalis, in children is challenging. JAK inhibitors, baricitinib,target JAK1/2, and ritlecitinib, target JAK3/TEC have shown promise in treating severe cases of AA in adolescents and preadolescent in clinical trials (2).

## Case report

An 11-year-old girl presented with extensive scalp hair loss that had persisted for two years, with complete loss of scalp hair but preservation of her eyebrows and eyelashes. She was diagnosed with alopecia totalis, which significantly impacted her quality of life, leading to social withdrawal and decreased self-esteem. She also suffered from allergic rhinitis for 2 years. Thyroid-stimulating hormone, FT3, FT4, anti-nuclear antibody, and anti-double-stranded DNA antibody were found to be in the normal range.

**Figure 1:**
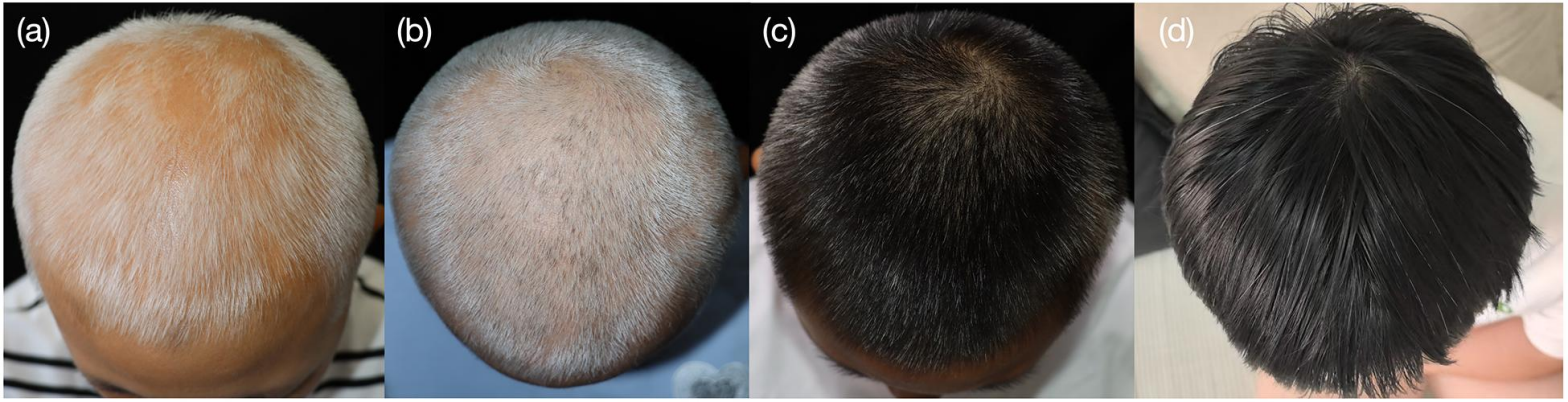
Clinical photographs demonstrating the sequential treatment response and pigment recovery in an 11-year-old preadolescent with alopecia totalis. **(a)** After 7 months of baricitinib therapy (2 mg/d combined with topical halomethasone): predominantly white and gray hair regrowth with sparse black hairs, SALT 65%. **(b)** Six weeks after switching to ritlecitinib (50 mg/d): emerging black hairs interspersed among white and gray regrowth, indicating early pigment restoration. **(c)** Six months after ritlecitinib initiation: complete scalp hair regrowth with near-complete repigmentation, SALT 0%. **(d)**Twelve-month follow-up on ritlecitinib: sustained complete regrowth with stable pigmentation and normal hair density, SALT 0%.

**Figure 2:**
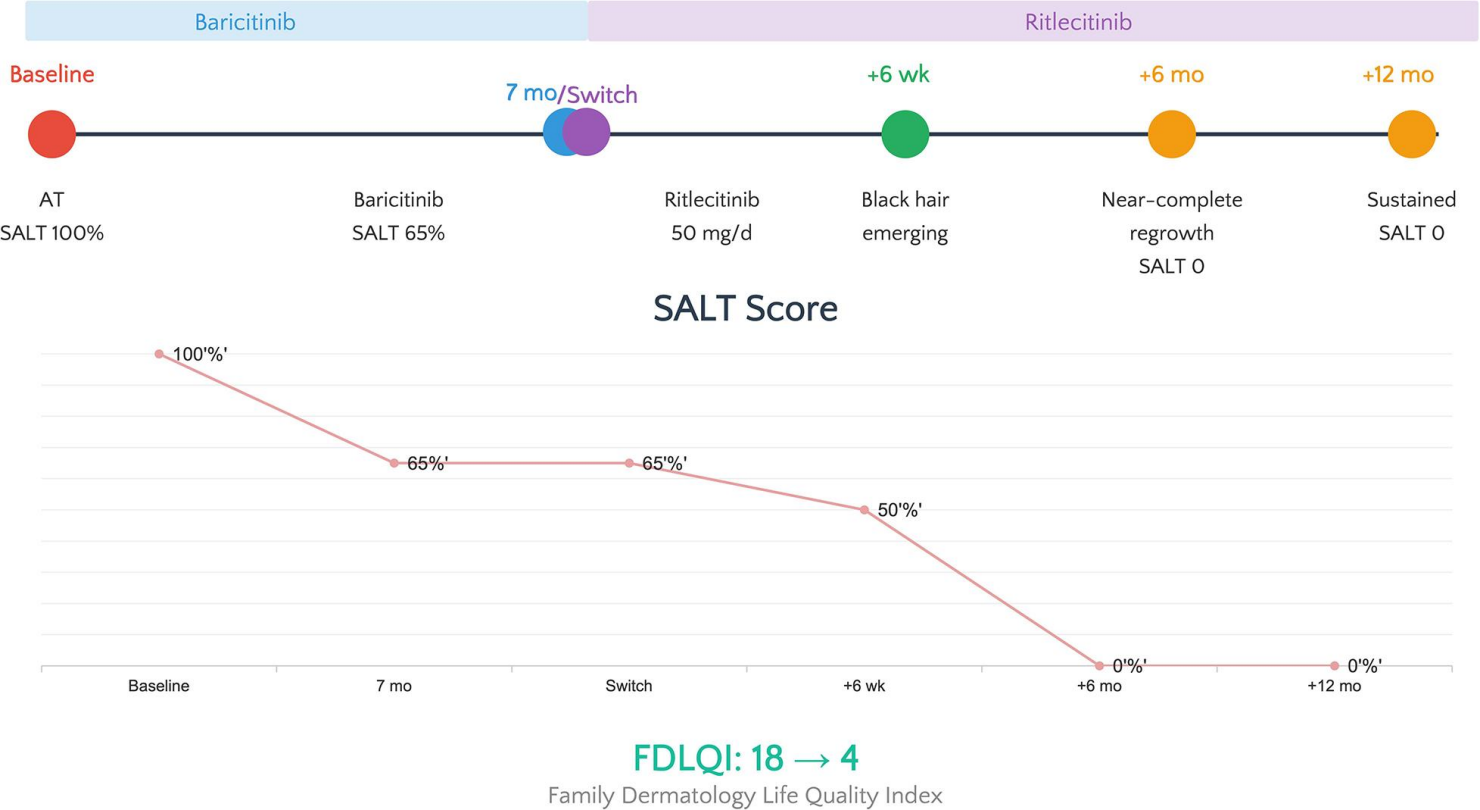
Treatment timeline.

She initially received baricitinib and topical halomethasone cream for seven months, resulting in the regrowth of white and gray hairs, with a SALT (Severity of Alopecia Tool) score of 65%. However, black hair remained sparse. The switch from baricitinib to ritlecitinib was prompted by incomplete response—after 7 months of baricitinib 2 mg/d combined with topical halomethasone, the patient achieved SALT 65% with predominantly white and gray hair regrowth, but black hair remained sparse. Given the patient's significant quality of life impairment and the goal of complete aesthetic recovery, we opted for ritlecitinib based on its distinct mechanism targeting JAK3/TEC kinase, which may offer differential immunomodulation in the hair follicle microenvironment (3).The patient had no notable personal or family history of vitiligo. After discontinuing previous treatments, ritlecitinib was prescribed at 50 mg/day. Six weeks later, a few black and gray hairs were observed, and by six months, there was complete hair regrowth and repigmentation (Figures 1, 2). Family Dermatology Life Quality Index (FDLQI) is a validated quality-of-life instrument specifically designed to assess the impact of skin disease on family functioning, with scores ranging from 0 to 30, where higher scores indicate greater impairment,The FDLQI of the case decreased from 18 to 4 (Figure 2). The patient was regularly monitored with monthly follow-up visits. No significant side effects were reported during treatment with ritlecitinib, and laboratory tests, including liver function and complete blood count, remained within normal limits.At the 12-month follow-up visit, the patient maintained complete hair regrowth with stable pigmentation (SALT 0). No signs of relapse or disease recurrence were observed. Laboratory monitoring, including liver function tests, complete blood count, and lipid profile, remained within normal limits. The patient reported sustained improvement in quality of life with no treatment-related adverse events.

## Discussion

Alopecia areata (AA) is an immune-mediated disorder resulting from the collapse of hair follicle immune privilege. In addition to keratinocyte dysfunction, immune-mediated injury to melanocytes within the hair bulb is a well-recognized feature of the disease. Clinically, this is often reflected by the regrowth of depigmented or gray hair during the recovery phase. Although hair regrowth is commonly used as the primary therapeutic endpoint, the restoration of hair pigmentation represents a more complex and less well-characterized aspect of follicular recovery.

Herein, we report a rare and instructive case of an 11-year-old preadolescent with alopecia totalis who achieved complete hair regrowth with simultaneous white-to-black hair conversion following sequential JAK inhibitor therapy. The prolonged follow-up (12 months on ritlecitinib, 19 months total JAK inhibition) and the detailed documentation of the pigment recovery timeline provide valuable real-world evidence for this underreported phenomenon in pediatric severe AA.

The regrowth of white hairs and pigment regression following alopecia areata (AA) exhibit significant clinical heterogeneity. From the overnight white hair formation during the acute phase (Marie Antoinette syndrome) (4), to years of depigmented regrowth during the recovery phase, and finally to complete pigment restoration in this case following JAK inhibitor treatment, these manifestations reflect varying degrees of immune-mediated damage to hair follicle melanocytes in AA.

Previous studies have demonstrated that the repigmentation of white hair after alopecia areata (AA) is highly dependent on the duration of immunosuppression. Dinh et al. reported a case of a 62-year-old patient with diffuse AA who achieved hair regrowth after a 6-week short-course prednisolone treatment but remained completely white six months later (5).Wade and Sinclair reported a 17-year-old patient who, without systemic immunosuppression, exhibited persistent occipital gray hair that did not turn black over a period of 7 years (6). In contrast, Tian et al. recently reported two cases of persistent hair regrowth in patients who underwent long-term treatment with methylprednisolone combined with compound glycyrrhizinate for 2 to3 years, achieving complete transition from total white hair to normal black hair (7). However, current evidence is largely limited to case reports and small series, and the relationship between treatment duration and repigmentation remains to be fully elucidated.

In the present case, the patient initially exhibited regrowth of predominantly white and gray hair during treatment with baricitinib (SALT 65% at 7 months), followed by emergence of black hair at 6 weeks and complete hair regrowth with near-complete repigmentation at 6 months after switching to ritlecitinib. At 12-month follow-up, sustained SALT 0 and stable pigmentation were maintained. While a causal relationship cannot be definitively established in a single case, the temporal association between prolonged JAK inhibitor therapy and pigment restoration is noteworthy. The patient exhibited predominantly white and gray regrowth during 7 months of baricitinib, followed by progressive darkening over 12 months of ritlecitinib, achieving sustained SALT 0 with stable pigmentation. This pattern is most consistent with a time-dependent recovery of melanocyte function during prolonged JAK inhibition, though whether this reflects a class effect of JAK inhibitors or the natural disease course remains uncertain. The consistent monthly improvement pattern and absence of prior spontaneous repigmentation during 2 years of untreated disease support a treatment-associated contribution, though confounding factors cannot be fully excluded.

Moreover, compared with conventional systemic corticosteroids, JAK inhibitors provide more targeted immunomodulation (8), although direct comparative efficacy and long-term outcomes, especially in pediatric populations, remain under investigation (9). In the present case, the patient initially exhibited regrowth of predominantly white and gray hair during treatment with baricitinib, followed by complete hair regrowth with near-complete repigmentation after switching to ritlecitinib. While a causal relationship cannot be definitively established in a single case, the temporal association suggests a potential role of sustained JAK inhibition in facilitating both hair regrowth and pigment restoration.

Mechanistically, JAK inhibitors may promote melanocyte recovery by suppressing inflammatory pathways, including interferon-*γ*–mediated signaling, thereby creating a more favorable microenvironment for melanocyte stem cell (MSC) regeneration (10). However, treatment responses are not uniform. Lawati Limbu et al. reported a patient with alopecia totalis who developed persistent white hair despite baricitinib therapy (11), indicating variability in pigment recovery. Due to the clinical nature of this report, histological or molecular validation of melanocyte recovery was not performed. Future studies incorporating hair follicle biopsy and immunohistochemistry for melanocyte markers (e.g., SOX10, MITF) are needed to confirm the proposed mechanisms.

In our case, sparse black hair emerged 6 weeks after the patient switched to ritlecitinib, and complete hair repigmentation was observed at 6 months. Notably, the patient had previously received baritinib treatment. Given the sequential use of two JAK inhibitors, we cannot confirm whether the pigment recovery was driven by baritinib or ritlecitinib. This clinical manifestation is in line with the phenomenon that pigment recovery lags behind hair regrowth, as reported by Tian et al. (7). Such a time lag may be attributed to the biological process that requires a full hair cycle for melanocyte stem cells (MeSCs) to migrate from the hair papilla to the hair bulb and complete functional reconstruction.

In contrast to vitiligo-associated leukotrichia, where melanocyte stem cells are often depleted, depigmented hair in alopecia areata is generally considered reversible, reflecting functional impairment rather than permanent loss of melanocytes.

From a mechanistic perspective, during the pathogenesis of alopecia areata, the disruption of the hair follicle immune microenvironment not only attacks melanocytes in the bulb but may also affect the homeostasis of melanocyte stem cells (MSCs)in the follicular protrusion.Yamaguchi et al. reviewed that hair regrowth may occur within 4 weeks of treatment, while pigment restoration typically requires 4–6 months or longer (9).This time lag suggests that although JAK inhibitors can alleviate immune attacks and promote hair growth, the activation, migration, and functional recovery of melanocyte stem cells (MSCs) require a longer period of immune microenvironment improvement.

At 12-month follow-up, the sustained SALT 0 and stable pigmentation observed in this case support the durability of follicular recovery following prolonged JAK inhibition. Long-term follow-up beyond 12 months is ongoing to assess the durability of response and potential for relapse. Potential confounders including seasonal variation, concurrent topical therapy, and natural disease fluctuation were considered. The consistent temporal relationship between treatment initiation and pigment recovery, combined with the absence of prior spontaneous repigmentation during 2 years of disease duration, supports a treatment-associated effect, though definitive causality requires controlled studies.

In this case, complete hair regrowth and repigmentation were achieved after prolonged JAK inhibitor therapy, suggesting that sustained treatment may be important for restoration of follicular function. However, these findings should be interpreted with caution given the single-case design, and further studies are needed to better define the mechanisms and predictors of pigment recovery in AA.

## Conclusions

JAK inhibitors have emerged as effective therapeutic options for severe pediatric alopecia areata; however, repigmentation of white hair during treatment remains rarely reported with limited. evidence.In this study, we reported an 11-year-old preadolescent with alopecia totalis who achieved complete hair regrowth and repigmentation following prolonged therapy. The patient has now completed 12 months of ritlecitinib therapy with sustained SALT 0 and stable pigmentation, supporting the durability of response. The patient showed no adverse effects during treatment. These findings contribute valuable evidence to the limited literature on JAK inhibitor use in preadolescent patients with severe alopecia areata, though generalizability is limited by the single-case design.

## Data Availability

The original contributions presented in the study are included in the article/Supplementary Material, further inquiries can be directed to the corresponding author.
